# To what extent are the wishes of a signatory reflected in their advance directive: a qualitative analysis

**DOI:** 10.1186/1472-6939-15-52

**Published:** 2014-06-30

**Authors:** Friedemann Nauck, Matthias Becker, Claudius King, Lukas Radbruch, Raymond Voltz, Birgit Jaspers

**Affiliations:** 1 Clinic for Palliative Medicine, University Medical Centre, Robert-Koch-Str. 40, 37075 Göttingen, Germany; 2 HAWK Hildesheim/Holzminden/Göttingen, University of Applied Sciences and Arts, Faculty of Social Work and Health, Brühl 20, R. 03, 31134 Hildesheim, Germany; 3 Department of Palliative Medicine, University Hospital Bonn, Sigmund-Freud-Str. 25, 53127 Bonn, Germany; 4 Department of Palliative Medicine, University Hospital Cologne, Dr.-Mildred-Scheel House, Kerpener Straße 62, 50937 Cologne, Germany

**Keywords:** Advance directive, Patient preferences, End-of-life care

## Abstract

**Background:**

Advance directives (ADs) are assumed to reflect the patients’ preferences, even if these are not clearly expressed. Research into whether this assumption is correct has been lacking. This study explores to what extent ADs reflect the true wishes of the signatories.

**Methods:**

Semi-structured interviews (INT), pretest. Transcribed INT and the contents of ADs were inductively categorised (Mayring) and triangulated. Software: MAXQDA 2007. Participants: Patients receiving palliative care (PPC), healthy (H) and chronically ill (CI) individuals with an AD completed ≥3 months prior to recruitment.

**Results:**

Between 08/2008 and 07/2009, 53 individuals (20 H, 17 CI, 16 PPC) were interviewed (mean age 63.2 years (55–70 years)), 34% male). Most important (in)consistencies between preferences as expressed in INT compared to ADs included preconditions for termination/rejection of life-sustaining measures, refusal of/demand for medical interventions and the nomination of proxies. Standardized AD forms were rarely tailored to the individual. We found a high tendency to use set phrases, such as *want to die with dignity* or *do not want to suffer/vegetate*. Likely events in the course of an existing progressive disease were not covered, even in ADs of PPC close to death.

**Conclusions:**

Only some of the incongruities between verbally expressed preferences and the contents of the AD can be put down to use of standardized forms or lack of medical knowledge. Nevertheless, the non-involvement of a doctor in the process of making an AD must be seen as potentially problematic and seeking medical advice should be promoted by politics and physicians. Standardised forms should encourage amendments and present space for free text entries for all aspects covered. Set phrases need to be defined by the individual to enable them to be translated into a specific course of action.

## Background

In ageing societies growing numbers of older adults will develop diseases that gradually impair their decision-making capacity [1,2]. Therefore, it is widely recommended that persons draw up advance directives (ADs) in which they discuss their treatment preferences or assign well-informed proxies in order to safeguard patient autonomy in critical situations when they are temporarily or no longer able to communicate these preferences.

The German Federal Supreme Court declared in 2003 that an advance directive (AD) must be respected and patients’ entitlement to refuse treatment not restricted to the dying phase. Some cases, in which the patient’s family or proxy went to court to fight for these rights, showed that neither clinical practice nor decisions taken in local German courts were consistently in accordance with this judgment [3]. Therefore, the need for an explicit legal regulation on ADs became subject to a long public, political, judicial and medical debate, resulting in new legislation enacted in 2009. This explicitly confirmed that, if the statements in an AD are clearly applicable to the clinical situation, refusal of treatment must be respected regardless of the nature or likely progression of the illness. Provisions were also made for people who lack capacity, where no valid or applicable AD exists. According to the legal regulations, in these cases ADs must be checked for important information about a person’s views which can be taken into account in the best interests decision making process, even if they are not binding. In case no AD exists, the probable will of the patient must be explored by gathering information: Did patients discuss preferences with relatives, friends or a proxy? What is known about their moral values or religious/spiritual viewpoint?

Unlike in other countries, for example Austria, the new German legislation did not make medical advice obligatory.

With these new regulations, the legislator wanted not only to strengthen patient rights but also promote legal instruments for patient self-determination, such as ADs, durable power of attorney etc. A representative survey had shown that the prevalence of ADs in Germany was about 11% in adults [4]; more recent representative data are not available.

It has been questioned, however, whether ADs are suited to facilitate end-of-life care in accordance with the true preferences of the signatory [5-7]. Furthermore, it has been suggested that ADs presuppose more control over future care than is realistic [5]. Medical crises cannot be predicted in detail [5]. ADs may be of questionable validity because signatories have a poor understanding of medical care and therefore unwittingly misrepresent their preferences [8].

ADs are also problematic in that preferences for end-of-life care are known to vary over time [9-11]. Furthermore, they can be influenced by the content, structure and underlying attitude of standard forms, depending on their source; for example, forms provided by the Catholic Church as compared to those from ‘right-to-die’ organizations [12].

Legal regulations governing ADs and recommendations for their use are, however, based on the hypothesis that they reflect the patients’ preferences, even if these are not clearly expressed. Research into this hypothesis has been lacking, so, in the context of a multi-centre research project, we wanted to explore to what extent ADs reflect the true wishes of the signatory using an open qualitative approach [13]. Further, we wanted to explore if consistencies and inconsistencies between what study participants believed to have addressed in their ADs and an analysis of the respective documents indicate relevant implications for clinical and non-clinical practice regarding ADs.

## Methods

### Design

Qualitative multi-centre study with inclusion of non-clinical participants.

### Participants and settings

Participants were recruited and interviewed between 08/2008 and 07/2009. Those without acute or chronic illness who claimed not to be taking any regular medication were allocated to group 1 (healthy persons). Those who claimed to be taking regular medication for a chronic illness qualified for group 2 (chronically ill persons). Group 1 and group 2 participants were recruited via local newspapers. Group 3 was formed from participants who suffered from a progressive incurable disease and were either inpatients or outpatients at one of the palliative care services of the collaborating university medical clinics in Bonn/Göttingen, Aachen and Cologne, Germany.

### Inclusion criteria

The study was limited to individuals aged between 55 and 70 years, in order to reflect the mean age of patients in palliative care units in Germany according to the nationwide core documentation system Hospice and Palliative Care Evaluation (HOPE) [14]. All participants were screened by use of the Mini Mental State Examination, the authorized German version of the test for grading the cognitive state of patients by the clinician developed by Folstein et al. (MMST-D) [15].

### Exclusion criteria

Excluded were individuals who did not meet the age group, with an AD ≤ 3 months, with a Mini Mental State ≤20, who showed signs of depression (Hospital Anxiety and Depression Scale – German version (HADS-D) [16] or with high symptom load (Minimal Documentation System MIDOS [17]).

### Procedure

Group 1 and group 2 participants: The adverts in local newspapers asked for potential participants in a study concerning ADs, age 55–70 and an AD completed at least 3 months ago. A hotline was open on two Saturdays, answered by three researchers who checked if these two inclusion criteria were met. If this was the case and the callers wanted to take part in the study after a more detailed explanation without giving away the research question, an interview was scheduled (standardized explanation: We are interested in ADs and would like to interview you about your treatment preferences and whishes for end-of-life care. Would you also agree to bring a copy for us to study?). After the analysis phase, all participants were invited – as announced during the encounters - to an informative meeting. At this meeting the researchers fed back anonymised results and problematic issues concerning ADs. Guest speakers were a lawyer and the head of an AD advice service; more general questions were answered in public, for individual advice participants could schedule meetings with the guest speakers (free of costs). This event was also planned on grounds of ethical obligation towards the study group.

Group 3 participants:

The palliative care services at the three collaborating university clinics were visited by one of the researchers from Bonn University and introduced to the study. Local researchers in Aachen, Cologne and Bonn regularly visited the services, checked newly admitted patients for the inclusion criteria age and AD. Of those, only patients that were estimated fit for research by the local clinical teams (considering symptom load, performance status, mental status) by the clinicians were approached by the local researcher in order to discuss participation. Those who wanted to participate were then asked to perform MMST and HADS-D measurements, supervised by the local researchers. If these criteria were met too, an interview was scheduled with the researcher from Bonn.

The study protocol was reviewed and approved by the Research and Ethics Committees of the Medical Association of North Rhine, Germany and by all the medical faculties involved (2006234, EK 11d6/07, 07–157). It was in accordance with the recommendations stated in the Helsinki Declaration of 1975. The written consent of all participants was obtained.

Data was managed according to the data protection law and participants were fully informed in person and in writing.

### Analyses

After developing a screening sheet and a semi-structured interview guide by an expert focus group, a pre-test in 07/2008 (n = 7) resulted in minor amendments to the interview guide (Table 1). Where applicable, i.e. after most questions, we asked: Did you address/describe this in you AD? The interviews were conducted between 08/2008 and 10/2009; fully transcribed according to standard transcription rules and analyzed applying an inductive category development. Encoding was carried out using the qualitative data analysis software MAXQDA 2007. ADs were searched for matching or contradictory statements as compared to the participant’s interview (triangulation) and their content was then inductively categorized under the pre-set main categories of congruence and incongruity [18]. Congruencies and incongruities of statements in the AD as compared to interview were categorized as either *non-specific* or *specific*. Non-specific was defined as without individualized situative/procedural context; specific as with individualized situative/procedural context.

**Table 1:** Interview guide

**Areas of interest**	**Questions**
Reason/motivation	1. Was there a special reason why you made an advance directive?
	Prompts:
	a. Can you tell me more about this reason?
	b. Did you make your advance directive before you were given this diagnosis or after?
	c. What do you hope for, now that you have an advance directive?
	d. Can you tell me the most important things you want to say?
Support/advice received/information of others	2. Did you seek assistance in making your advance directive?
	Prompts:
	a. (if yes) Who helped you?
	b. Why did you ask particularly this person?
	c. (if no doctor was mentioned) Did you also seek assistance from a doctor?
	3. Does your doctor know about your advance directive and its contents?
Applicability	4. Did you consider at which point in time you want others to act according to your advance directive?
	Prompts:
	a. (if yes) What do you have in mind? The very end of life, or situations where you are no longer able to express yourself or other?
	b. Did you express certain preferences in the context of particular (probable future/already existing) diseases?
	c. (if yes) Will these count throughout the course of the disease or just in certain phases?
	5. Do you want the contents of your advance directive to be applicable for emergencies or serious accidents?
Values	6. Are there values which have been of particular importance in your life?
	7. Are there values which you disapprove of?
	8. Did these values which are important for you have an influence on your advance directive?
	9. Do these values also determine your end-of-life preferences?
Treatment preferences/situative context/decision-making	10. What is of particular importance for you when it comes to the end of your life?
	11. Is there something that you would not wish to happen under any circumstances and that those who are acting on your advance directive should respect?
	12. Is there something that you would categorically wish to happen and that those acting on your advance directive should respect?
	13. Is this true for all situations you can think of?
	14. Are there situations or scenarios in which you would prefer that decisions are made by others rather than you?
Effect of having completed an AD	15. Does having an advance directive make you feel secure?
	16. Have you since been thinking more often about death and dying?
Amendments/Changes	17. Have you resigned your advance directive since you first made it?
	18. Have you changed it since first making it?
	Prompts:
	a. (if no) Why didn’t you change anything?
	b. (if yes) What did you change?
	c. Why did you change this?
	d. Did you seek assistance in making these changes?
	e. (if yes) From whom and why?
Proxy/durable power of attorney	19. Do you have a proxy?
	20. Why did/didn’t you appoint one?
	21. What do you expect from this durable power of attorney?
	22. Whom did you appoint as proxy?
	23. Why this person?
Closure	24. Thank you for your participation. How do you feel after this interview? Do you have any questions?

The use of set phrases from the same semantic field within a given context was categorized as *non-specific congruence.* The use of set phrases from differing semantic fields within a given context was categorized as *non-specific incongruity*.

**Figure 1:**
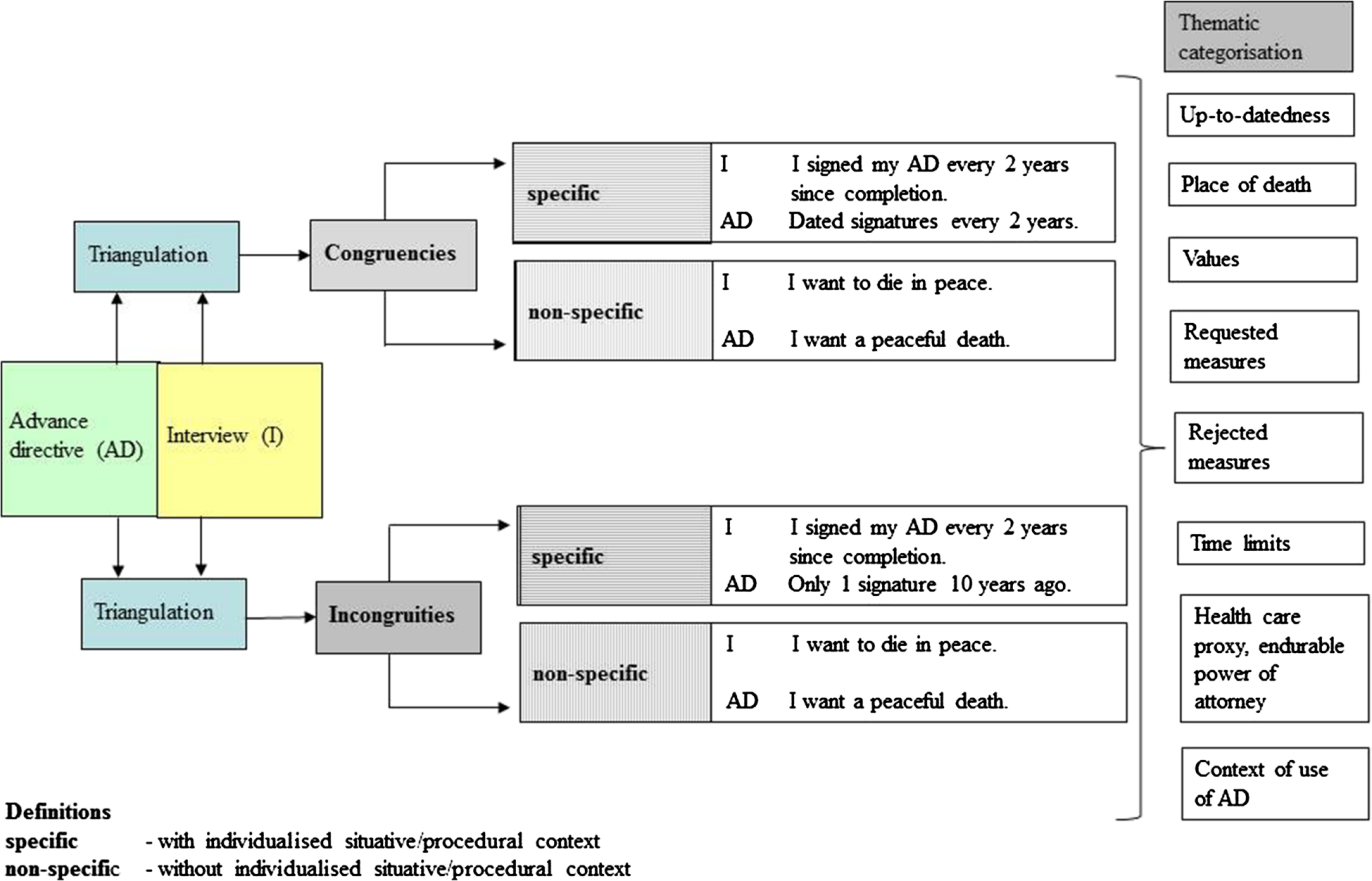
Structure of analysis.

Statements of fact or with unambiguous wording in a given context were classified as *specific congruence.* Conflicting factual or unambiguous statements were classified as *specific incongruity*. The structure of the analysis including clarifying examples is presented in Figure 1. The sample of findings of specific and non-specific congruencies and incongruities was then inductively categorized into thematic categories.

The documents themselves were also searched for ambiguous wording and inconsistencies. Interviews and ADs were analyzed independently by two researchers and the results discussed until a consensus was reached.

Deutsche Forschungsgemeinschaft (DFG) sponsored this study (German Research Foundation grant no. NA 780/1-1). The funders had no role in study design, data collection and analysis, decision to publish, or preparation of the manuscript.

## Results

Response rates: 37 of the 59 persons who called the hotline after having read the advert for group 1 and group 2 recruitments met the inclusion criteria; all 37 persons wanted to take part in the study. Of the 234 patients admitted to palliative care services at the three collaborating university clinics, 25 patients met the inclusion criteria age group and AD, were rated as fit enough for participation by the team and asked to participate. Of those, 5 did not want to take part. The remaining 20 persons passed the MMST und HADS-D measurements, but after inclusion 2 declined and 2 died. Therefore, a total of 53 persons were included in this study. All group 3 patients had advanced cancer (mostly of the lung and breast, but also cancer of the pancreas and prostate, colorectal, ovarian, appendicular and gastric cancer and liposarcoma). About one third had a performance status of ECOG 2 (37%), two thirds had ECOG 3.

Epidemiological information from group 2 was obtained by the participants themselves and was therefore partly ambiguous. Conditions included cardiovascular diseases, osteoarthritis, diabetes, fibromyalgia, liver cirrhosis, and other. Sociodemographic and interview/AD related data are presented in Tables 2 and 3 respectively.

**Table 2:** Sociodemographic data of all participants and by group (n; mean; SD, range; median;%)

**Sociodemographic data**				
	**All**	**Healthy**	**Chronically ill**	**Palliative patients**
	**(n = 53)**	**(n = 20)**	**(n = 17)**	**(n = 16)**
Age [years; mean; SD; range]	63.2 ± 4.4(55–70)	64.2 ± 4.2(55–70)	62.6 ± 4.2(55–70)	62.5 ± 4.4(55–70)
Median	63.0	65.0	63.0	62.0
Gender [female; n;%]	35 (66%)	13 (65%)	13 (77%)	9 (56%)
Marital status [married; n;%]	29 (55%)	11 (55%)	11 (65)	7 (44%)
No. of children [n; mean; SD; range]	1.5 ± 1.0(0–4)	1.6 ± 1.2(0–3)	1.4 ± .79(0–2)	1.5 ± 1.1(0–4)
Median	2.0	2.0	2.0	1.5
Living situation [≥two-person household; n;%]	34 (64%)	12 (60%)	10 (59%)	12 (75%)
Education [university degree; n;%]	21 (40%)	11 (55%)	7 (41%)	3 (19%)
Religious affiliation [n;%]				
Catholic	21 (40%)	4 (20%)	8 (47%)	9 (56%)
Protestant	11 (21%)	5 (25%)	4 (24%)	2 (13%)
Other	1 (2%)	0	0	1 (6%)
None	20 (38%)	11 (55%)	5 (29%)	4 (25%)
Self-rating of adherence to religious beliefs [numeric rating scale from 0 to 10; 0 = not at all, 10 = very much; mean]	5.0 ± 2.5(0–10)	4.9 ± 2.9(0–10)	5.1 ± 2.0(0–8)	5.1 ± 2.5(0–9)
Median	5.0	5.5	5.0	5.0

**Table 3:** Interview/advance directive related data of all participants and by group (n; mean; SD, range; median;%)

**Interview length and AD-related data**				
	**All**	**Healthy**	**Chronically ill**	**Palliative patients**
	**(n = 53)**	**(n = 20)**	**(n = 17)**	**(n = 16)**
Interview length [min; mean; SD, range];	22:48 ± 9:50(11:37–1:03:10)	26:33 ± 12:40(12:31–1:03:10)	22:42 ± 7:21(15:31–46:46)	16:14 ± 5:47(11:37–32:50)
Median	19:12	23:54	20:54	16:11
Proxy appointed [n;%]	48 (91%)	19 (95%)	16 (94%)	13 (81%)
Of those: family member as appointed proxy [n;%]	43 (90%)	16 (84%)	15 (94%)	12 (92%)
Physician to be notified named in AD [n;%]	9 (17%)	3 (15%)	2 (12%)	4 (25%)
Date of completion of the AD previous to interview [months; mean; SD, range]	43 ± 48.0(3–300)	49 ± 34.7(7–116)	58 ± 68.0(4–300)	20 ± 25.3(3–84)
Median	34.0	41.5	49.0	5.5
Modification of AD since first completion [n;%]	13 (25%)	6 (30%)	4 (24%)	3 (19%)
Medical advice (physician) [n;%]	2 (4%)	0 (0%)	1 (6%)	1 (6%)
Advice by notary/lawyer [n;%]	8 (15%)	0 (0%)	4 (23%)	4 (25%)

A quarter of all participants had changed parts of their AD at least once since it was first completed, and the ADs of palliative patients were completed more recently on average than those of the other groups.

Legal advice was sought significantly more often than that of a doctor.

Professional advice was sought by 12 participants (physician = 2, nurse = 1, lawyer/notary = 8, self-employed advisor = 1), another 8 participants included family members. In 17 cases, the physician knew the participant’s advance directive, 36 participants never told their doctor about its existence. Categories of reasons for seeking or foregoing advice were trust/lack of trust, autonomy, rejection and financial considerations.

More detailed methods and results regarding motivation for the completion of an AD and the advice obtained are published elsewhere [3,19].

Inductively identified sub-categories of specific and non-specific statements, including exemplary quotations/citations are shown in Table 4.

**Table 4:** Sub-categories of specific congruencies and incongruities

**Categories**	**Examples of incongruities**	
**Congruence/Incongruity**	**Statements in interviews**	**Findings in advance directives (AD)**
Up-to-datedness	“After completion, I regularly signed it to reconfirm its contents.” (24S)	No more than one signature, dating from the day of completion in 04/2004.
Place of death	“I want to die in a hospice or a palliative care unit.” (12D)	The preferred place of death, as stated in her AD, is her home.
Values	“I described the values that are important for me when it comes to dying and that they are based on my faith.” (47D)	Neither faith nor faith-based values were mentioned.
Requested/Wanted measures	“I want efficient pain control, no matter what happens.” (15 L)	The participant refuses any kind of measure or treatment.
Rejected measures	“I would not want to live without legs or with paraplegia. For these situations, I refuse any further treatment.” (2B)	AD addresses permanent unconsciousness, probable permanent brain damage, permanent failure of vital body functions but neither amputation nor paraplegia.
Time limits	“I am very clear about how long I would accept certain measures and have explained this in my AD. For example, I would accept a percutaneous feeding tube for no longer than 3 three weeks and then only if I will fully recover.” (14 W)	A time limit for a percutaneous feeding tube is not mentioned. AD states different time limits on different pages for resuscitation (e.g. no resuscitation after 7 min/3 min of cardiac arrest) and for discontinuation of certain measures in case of unconsciousness/coma (after a maximum of 3 months/3 weeks).
Healthcare proxy and endurable power of attorney	“My two sons are my proxies. They live nearby and know very well what my preferences are.” (21S)	Not her sons but her husband is named in the document. He had died three years ago.
Context of use of the AD (dying process, certain illness, emergency)	“If I am no longer able to speak, eat or swallow.” (16H)	The document is only valid for the dying process.

### Congruencies: non-specific

A large number of non-specific congruencies were found, mostly in the context of a qualitative description of the dying process [for n = 42] *(want a peaceful death, dignified death, good death; don’t want to suffer a wretched, miserable death)*, refusal of treatment *(no machines, no invasive treatment)* or concepts of quality of life *(don’t want to suffer, to vegetate)*.

### Incongruities: non-specific

Only a few non-specific incongruities were found, therefore no further sub-categorization was undertaken.

### Congruencies and incongruities: specific

Specific congruencies were found in the analyses of all participants. Group differences with regard to kind and frequency of congruencies were not detected.

Table 4 presents categories that were found for specific congruencies and incongruities. Examples of statements/facts are given only for incongruities because of their special relevance to the aim of this study.

Further sub-categories, only found for specific congruencies, were: holding hands (request for people to be present at time of death) and decision-making (instructions as to who should be involved). One category was found only to relate to incongruities: reference to already existing diseases. None of the participants who claimed to have written their AD with consideration of their disease had done so. Example: *“My AD focuses on treatment preferences regarding my COPD disease”*; a standard form was used in which COPD was not referred to (51 K).

Specific incongruities were found in the comparative analysis of interviews and ADs of 28 participants (group 1: 10 of 20; group 2: 11 of 17; group 3: 7 of 16 participants).

Information on AD forms is presented in Table 5.

**Table 5:** Sources used for the completion of an advance directive (n = 53 participants)

**Advance directive**	**n**
Individual’s wording only	2
Choice of a standardized form with checkboxes and fields for free text entries	25
Choice of a standardized form with checkboxes and fields for free text entries, complemented with individually worded paragraphs	1
Compilation of parts from various standardized forms	17
Use of a form prepared by a legal professional who was contacted for advice	8

Standardized forms provided by organizations or sections taken from such forms were hardly ever changed by the participants. In a very few cases, sentences or paragraphs were deleted; additional remarks were inserted by only one participant.

Furthermore, fields for providing additional information were hardly used and if so, were filled with non-specific phrases, such as *I want to die with dignity*. One participant produced an AD consisting of three complete prepared forms from different sources, so as to further demonstrate its importance, but each form was fundamentally inconsistent with the others.

Legally prepared ADs consisted solely of text modules which could be downloaded free of charge from the internet. These were amended by neither the respective paid lawyers/notaries nor the participants. Due to the use of text modules from different forms and a lack of personalization, legally prepared ADs showed a high tendency to internal inconsistencies, as reported elsewhere [3].

Many ADs (n = 39) included a desire for pain control, but usually only during the dying phase.

*When two physicians independently diagnosed that I have irreversibly entered the dying phase I want to be given sufficiently dosed pain medication, even if this will shorten my life.* (14 J)

## Discussion

There are manifold definitions of the term AD or living will. It can be assumed that the underlying hypothesis of all of them is that these documents are a means of providing guidance for medical and healthcare decisions according to the true wishes and preferences of the signatory. Previous research on ADs has, for example, explored whether or not people would stick to what they had written [20] but not to what extent they accurately reflect the wishes of the signatories. We wanted to explore this gap, particularly because German legislation had recently sought to introduce explicit regulation of ADs.

Findings of specific incongruities in more than half of the participants’ ADs show that these documents may misrepresent their wishes. Whereas some, e.g. date of the last signature, are not likely to have an impact on an AD’s relevance in clinical practice, others may well be misdirecting end-of-life care, as in the examples given in Table 4 for the categories rejected measures and time limits.

Most participants stated that they had read their AD carefully prior to interview. Nevertheless, they failed to spot incongruities that could, in theory, be detected without medical knowledge. This was despite their self-selection for the study, which would imply a deep personal interest in the subject [21]. Most problematic were incongruities involving requests regarding particular measures or time limits. On the basis of the forms presented, it can be surmised that signatories had a poor knowledge of particular clinical situations and the relevance of potential measures. A study undertaken by Thorevska et al. in 2004 showed poor understanding of life-sustaining therapies in patients with living wills [8]. Most failed to involve doctors in making directives, as was also found to be the case in our three study groups Among the reported reasons for non-involvement of a physician in these groups were lack of trust in physicians and refusal of physicians to help with making an AD, but also the wish to complete this task autonomously. The results regarding matters of advice are presented and discussed in detail in a separate paper [3].

Accepting or rejecting life-sustaining measures via a tick list, before discussing the prognosis or purpose of care, was said to be putting the cart before the horse [21]. This may be confirmed by an interesting aspect of our findings; neither chronically ill nor palliative care patients mentioned their existing disease in their AD or went beyond considering commonly feared scenarios such as artificial nutrition/hydration or being in pain or a coma. Probable end-of-life scenarios relating to their disease were not discussed. Interestingly, phrasing of preferences regarding pain control in standardized forms implied that drugs taken to control pain may shorten life, a phenomenon also found throughout other ADs. The reason for this is not clear, but may be ascribed to the prevalence of myths relating to the use of strong opioids.

There was also a noticeable occurrence of non-specific congruencies, which at first sight may be taken as a positive outcome. The nature of these congruencies by our definition does, however, leave the need for interpretation and translation into particular courses of action that will fulfill the signatory’s expectations [22]. Set phrases such as *to die with dignity/in peace* are subject to individual interpretations and concepts and the difficulty in understanding non-specific phrasing was shown in a study by Porensky and Carpenter [23].

The tendency to use non-specific wording is theoretically less problematic where there is a proxy, but there is evidence that proxies make wrong assumptions. Shalowitz et al. reviewed studies with about 3,000 patient/proxy pairs and found differences in understanding in about a third of the cases [24]. Another study showed an overall surrogate positive predictive value for a high-risk scenario of 79.7% [25].

It follows that the fact that most of our participants had assigned a proxy cannot be seen as a reliable way of overcoming the flaws of imprecise wording. Most of the proxies were family members and in touch with the signatories on a regular basis, but according to Clements it is common for proxies to be badly informed [26]. The design of our study did not include exploring the extent to which proxies’ interpretations matched participants’ intentions, but that some participants had assigned a proxy who did not even know about his appointment, had never seen the AD nor knew about its existence, where it was filed or the signatory’s preferences for end-of-life care, must be seen as a concern. In some cases a family member was chosen as a proxy mostly to conform to societal norms, even though the signatory assumed that this person would not be able to cope with their preferences for discontinuation or refusal of treatment.

We had chosen the group divisions because it was being argued in public and political debate at that time that knowing about an already existing chronic illness may improve clarity and individualization of ADs. But group differences with regard to specific and non-specific congruencies/incongruities were not found. Instead, we identified some group-specific tendencies relating to the phenomenon of incongruity.

In both healthy individuals and palliative care patients, the motivation behind completing an AD had a clear impact on the way the document was conceived, as described elsewhere [19]. Healthy people often construed their AD as a kind of reversal of their bad experience of a loved one’s end-of-life care, these were often inconsistent with or irrelevant to their own situation.

Palliative care patients clearly expressed altruistic reasons for the contents of their AD. The reported motive for rejection of treatment or preference for dying somewhere other than home, was to make decisions easier for the physicians and not to be a burden to others, particularly family members. This tended to be at odds with their own personal values which stressed the importance of providing a high level of care for others. According to German law, ADs must be particularly carefully interpreted with regard to personal values, when the scenarios outlined for treatment decisions in the document differ from the actual situation in which a decision has to be made. Therefore we believe it is an important finding of this study that it cannot be concluded by implication that personal values in life give a definite indication of a person’s probable preferences for end-of-life care.

Since the time of study, problems arising from ambiguous phrasing in ADs and, for example, the clinically observed prevalence of ADs that were not applicable to the situations in question, have lead to the promotion of advanced care planning (ACP). Unlike in other countries, ACP, defined as systematic approach to ensure that effective advance directives (ADs) are developed and respected, is a rather new and not yet well-established concept in Germany. First studies showed promising results [27].

### Limitations and strengths of the study

The allocation of participants into groups of healthy and chronically ill patients was based on self-reported data which could not be triangulated with medical records. These groups showed a higher percentage of people with university degrees than group 3 and than the same age group in the general population in Germany. Self-selection can be seen as a flaw of the study, but may also (particularly because of this high level of education in our sample) actually add weight to our findings, given the likelihood that participants may be comparatively well informed about ADs. The responses, particularly from group 3 participants, may be biased by social desirability. The latter also had the shortest average interview length and may not have presented relevant issues as exhaustively as the other two groups. Another limitation may be the selected age group. We did this for reasons explained in the paper, but the inclusion of more age groups may have brought a wider variety of findings. It may be questioned that participants reported on their current care preferences which may be different from what they presented in their AD. To avoid this, we particularly asked if the aspects reported in the interview were also put down in writing.

A strength of the study design is the triangulation of interviews with persons who had completed an AD and the existing documents. To the best of our knowledge, this design has not been used before. The qualitative approach thus strengthened clinical observations. The inclusion of the three different participant groups facilitated insights in the matters of research also in non-clinical participants. This is important because drafting of ADs is an issue that concerns the general public. Another strength of the study design is that we considered the moral obligation to feed back problematic issues to the participants by organizing an informative meeting including the opportunity to obtain advice on their ADs free of costs.

## Conclusions

The results of this qualitative approach showed poor individualization and many incongruities of various kinds between verbally expressed preferences and the content of the AD.

This can be only partly put down to use of standardized forms or lack of medical knowledge.

Even though it is generally recommended to individualize information in an AD, a lack of such information might not be problematic. Firstly, many individuals may never find themselves in a situation where their AD becomes relevant to their clinical care. Secondly, patients with close contact to treating physicians, such as patients receiving palliative care or chronically ill patients, may discuss these issues in person and rely on documentation of their current preferences in patient charts. Patients may have a well-informed proxy. The presentation of our findings concentrated on incongruities and pitfalls in order to raise awareness about potentially important issues in the context of making or amending an AD.

We have reason to conclude that the non-involvement of a doctor in the process of making an AD must be seen as potentially problematic. The legislator based their decision not to make medical advice mandatory on conflicting laws regarding autonomy. It is true that a patient who can still communicate treatment preferences is legally entitled to rigorous refusal of life-preserving measures despite lack of understanding of the medical situation or on grounds of what is addressed by the German Federal Court as “unreasonable decisions”. In these cases, however, physicians (and others, e.g. family) can still seek to discuss matters more thoroughly with the respective patients. Such discussions cannot be initiated when a person is no longer able to communicate appropriately and an applicable AD has to be taken at face value. Therefore, we would like to recommend that seeking medical advice for statements in ADs should be promoted by politics (including a more sufficient remuneration for such services) and proactively offered by physicians. Also, ACP programs should be promoted.

Further, it seems necessary to raise awareness that set phrases, such as *to die with dignity* need to be tailored to the individual to enable translation into a particular course of action. Advice on ADs should include this important issue.

Standardized forms should provide space for free text entries in all sections of the documents, ask specifically for treatment preferences of diagnosed diseases in critical and/or end-of-life situations. Also, introductions to these forms should encourage users to add information beyond presented categories or to cross out categories that do not apply to their views and needs.

## Abbreviations

AD: Advance directive; COPD: Chronic obstructive pulmonary disease; DFG: Deutsche Forschungsgemeinschaft; ECOG: Eastern cooperative oncology group; HADS-D: Hospital anxiety and depression scale – German version; MIDOS: Minimal documentation system; MMST-D: Mini mental state examination - German version; HOPE: Hospice and palliative care evaluation.

## Competing interests

All researchers declare their independence from funders and sponsors. All authors declare that they have no competing interests.

## Authors’ contributions

FN: Study design, literature search, data analysis and interpretation, drafting and revising the manuscript. MB: Literature search, data collection, data analysis and interpretation, tables. CK: Literature search, data collection, data analysis and interpretation, tables. LR: Data analysis and interpretation, revising the manuscript. RV: Data analysis and interpretation, revising the manuscript. BJ: Study design, literature search, data analysis and interpretation, tables, drafting and revising the manuscript. FN and BJ contributed equally to the manuscript. All authors read and approved the final manuscript.

## Pre-publication history

The pre-publication history for this paper can be accessed here:

http://www.biomedcentral.com/1472-6939/15/52/prepub
